# Self assembled cationic cell penetrating peptide nanoparticles as efficient cargoes for antimicrobial agents

**DOI:** 10.1186/1471-2334-14-S3-P19

**Published:** 2014-05-27

**Authors:** A Aravind, A Deepu, K Santhosh Kumar

**Affiliations:** 1 Chemical Biology Lab, Rajiv Gandhi Centre for Biotechnology, Thiruvananthapuram, India

## Background

The development of technologies that enable bioactive molecules with low membrane permeability to effectively penetrate biological membranes is one of the greatest challenges in the pharmaceutical field. The HIV-1 transactivating transcriptor (TAT) peptide is one of the most widely used molecular beacons for drug delivery. Cationic antimicrobial peptides have received increasing attention due to their broad spectrum activities and ability to combat multi drug resistant microbes. Our aim is to develop cationic cell penetrating peptide nanoparticles which could efficiently deliver antimicrobial agents against infectious diseases.

## Methods

The cationic TAT peptides were synthesized by Fmoc solid phase peptide synthesis and were purified by semi preparative RP-HPLC. The molecular mass of peptides was confirmed by MALDI TOF-MS. The purified peptides were modified into self assembled nanoparticles (micelles) and structural characterization was done by a particle size analyzer and TEM. The antimicrobial activity evaluation was done against both Gram positive and Gram negative bacterial strains.

## Results

We have designed and synthesized arginine and lysine rich cationic TAT peptides and modified them into nanoparticles. These nanoparticles were structurally and functionally characterized. Some analogues of these nanoparticles cargoes showed significant antimicrobial activity against Gram positive and negative bacterial strains especially those cause brain infections with low MIC values. These were shown relatively low haemolysis.

## Conclusion

The findings of this study revealed the improved antimicrobial activity of modified cationic TAT peptide nanoparticles against different bacterial strains. This could revolutionize in the field of chemotherapy and can be efficiently used to treat brain infections and other diseases.

